# Detection Wavelength Control of Uncooled Infrared Sensors Using Two-Dimensional Lattice Plasmonic Absorbers †

**DOI:** 10.3390/s150613660

**Published:** 2015-06-10

**Authors:** Yousuke Takagawa, Shinpei Ogawa, Masafumi Kimata

**Affiliations:** 1 College of Science and Engineering, Ritsumeikan University, 1-1-1 Noji-Higashi, Kusatsu, Shiga 525-8577, Japan; E-Mail: Takagawa.Yosuke@ds.MitsubishiElectric.co.jp; 2 Advanced Technology R&D Center, Mitsubishi Electric Corporation, 8-1-1 Tsukaguchi-Honmachi, Amagasaki, Hyogo 661-8661, Japan

**Keywords:** uncooled infrared sensors, plasmonics, metamaterial, wavelength-selective

## Abstract

Wavelength-selective uncooled infrared (IR) sensors are highly promising for a wide range of applications, such as fire detection, gas analysis and biomedical analysis. We have recently developed wavelength-selective uncooled IR sensors using square lattice two-dimensional plasmonic absorbers (2-D PLAs). The PLAs consist of a periodic 2-D lattice of Au-based dimples, which allow photons to be manipulated using surface plasmon modes. In the present study, a detailed investigation into control of the detection wavelength was conducted by varying the PLA lattice structure. A comparison was made between wavelength-selective uncooled IR sensors with triangular and square PLA lattices that were fabricated using complementary metal oxide semiconductor and micromachining techniques. Selective enhancement of the responsivity could be achieved, and the detection wavelength for the triangular lattice was shorter than that for the square lattice. The results indicate that the detection wavelength is determined by the reciprocal-lattice vector for the PLAs. The ability to control the detection wavelength in this manner enables the application of such PLAs to many types of thermal IR sensors. The results obtained here represent an important step towards multi-color imaging in the IR region.

## 1. Introduction

Uncooled infrared (IR) sensors detect IR radiation from objects by converting thermal energy to an electrical signal [1]. Microelectromechanical system (MEMS)-based uncooled IR sensors typically consist of IR absorbers and temperature sensors with a thermal isolation structure where arrays of these can function as image sensors. Recently, there has been increased interest in such uncooled IR sensors due to significant technical progress and a growing range of applications, such as remote sensing, biomedical devices, security, fire-fighting, car sensors and home appliances [2,3].

We are developing an advance uncooled IR sensor with object discrimination as its function by the use of wavelength-selective absorbers based on plasmonics [4,5,6] and metamaterials [7,8,9]. Wavelength-selective uncooled IR sensors have significant potential because objects can be identified from their IR radiation spectrum [10]. For instance, gases can be identified from their absorption wavelengths [2]. There are many methods to realize wavelength-selective functions for filters [11,12], optical resonant structures [13], and multi-layer structures [14,15]. However, these methods require additional optical systems or complicated pixel structures, which lead to increased cost and prevent the establishment of arrays for use in image sensors.

Recently, we have developed wavelength or polarization-selective uncooled IR sensors using square lattice two-dimensional plasmonic absorbers (2-D PLAs) [16,17,18] and have also developed 3-D PLAs [19,20,21]. 2-D PLAs have an Au-based 2-D periodic dimple-array, where surface plasmon resonance (SPR) occurs. We have also demonstrated that triangular lattice 2-D PLAs can realize wavelength-selective detection [22]. However, the detailed effects of the lattice structures on the detection wavelength have not yet been studied. Here, we conduct a detailed investigation into the detection wavelength control of uncooled IR sensors according to the surface lattice structures of 2-D PLAs.

## 2. Design

**Figure 1:**
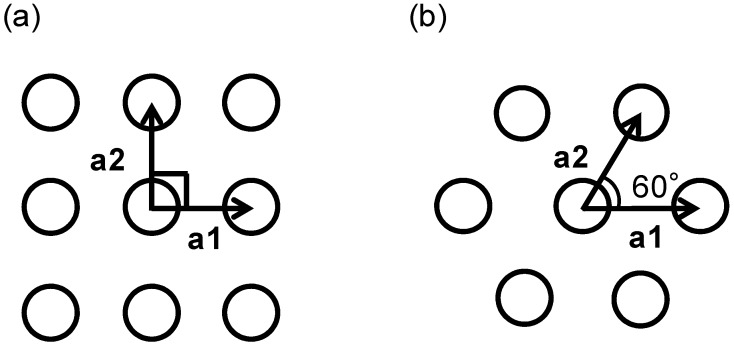
2-D lattice structure and primitive lattice vectors for (**a**) square and (**b**) triangular lattices.

Absorption wavelength control according to the 2-D lattice structure was theoretically investigated for both the square and triangular lattices. Figure 1a,b show schematic representations of the square and triangular 2-D lattices, respectively, where a1 and a2 are the primitive lattice vectors. The period (*p*) is the same for both structures.

SPR can be excited when it matches the following momentum equation [23]:

(1) $$\vec{K_{sp}}=\vec{K_{0}}\sin\theta \pm m\vec{G_{1}}\pm n\vec{G_{2}}$$

where $\vec{K_{sp}}$ and $\vec{K_{0}}$ are the surface plasmon wavevector and the incident light wavevector, respectively, θ is the incident angle, and $\vec{G_{1}}$ and $\vec{G_{2}}$ are the set of primitive reciprocal lattice vectors in the 2-D lattice. *m* and *n* are a set of integers.

We consider the normal incidence, *i.e.*, θ=0°:

(2) $$\vec{K_{sp}}=\pm m\vec{G_{1}}\pm n\vec{G_{2}}$$

The main $\vec{K_{sp}}$ can be obtained when Equation (3) is satisfied:

(3) $$\left|\vec{K_{sp}}\right|=\left|{\vec{G}}_{1(2)}\right|$$

and by consideration of the IR wavelength region [24]:

(4) $$\left|\vec{K_{sp}}\right|≅\frac{2\pi }{\lambda_{sp}}$$

where $\lambda_{sp}$ is the SPR wavelength:

(5) $$\lambda_{sp}≅\frac{2\pi }{\left|\vec{G}\right|}$$

The main SPR wavelength that corresponds to the main absorption wavelength in the square lattice 2-D PLA can be calculated as follows:

(6) $$\left|{\vec{G}}_{1}\right|=\left|{\vec{G}}_{2}\right|=\frac{2\pi }{p}$$

(7) $$\lambda_{sp}≅p$$

The main absorption wavelength is almost equal to the surface period, which coincides with the measurement results [17]. 

Next, the triangular lattice structure was calculated as follows:

(8) $$\left|{\vec{G}}_{1}\right|=\left|{\vec{G}}_{2}\right|=\frac{2}{\sqrt{3}}\cdot \frac{2\pi }{p}$$

The main SPR wavelength, which is the main absorption wavelength in the triangular lattice, can be obtained as follows:

(9) $$\lambda_{sp}≅\frac{\sqrt{3}}{2}\cdot p$$

The main absorption wavelength is expected to be shorter than that of the square lattice 2-D PLA. Theoretical investigations show that the primitive reciprocal lattice vector of the 2-D lattice structure is the main parameter controlling the absorption wavelength. 

**Figure 2:**
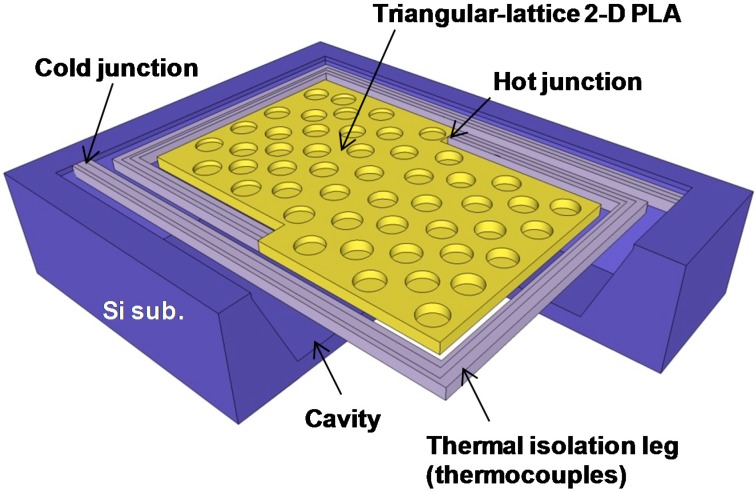
Schematic of the MEMS-based thermopile with a triangular lattice 2-D PLA.

Figure 2 shows the structure of the thermopile with a triangular lattice 2-D PLA used in this study. The thermopile was used as an uncooled IR sensor. The thermal isolation legs contain thermocouples that transform the temperature difference between hot and cold junctions into an electrical signal based on the Seebeck effect [25]. The absorber is thermally isolated using a cavity structure and a periodic structure is formed on the absorber surface. 

## 3. Sensor Fabrication

**Figure 3:**
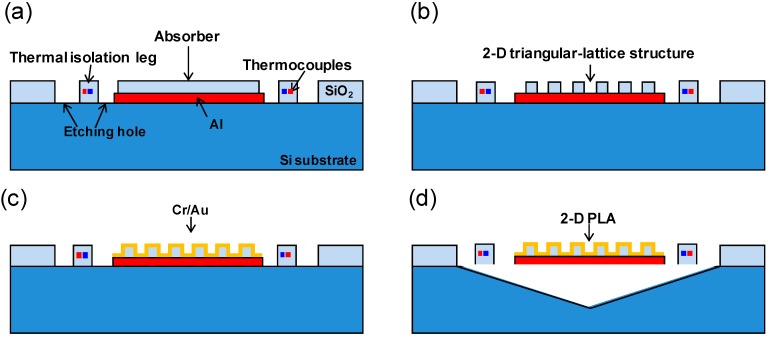
Procedure for the fabrication of a thermopile with triangular lattice 2-D PLAs. (**a**) Devices are formed on the surface of a Si wafer by a standard CMOS process. Etching holes are formed by RIE; (**b**) The 2-D triangular-lattice structures are formed on SiO_2_ using a dry etching process; (**c**) Cr/Au layers are sputtered; (**d**) The cavity is formed by TMAH etching.

Thermopiles were fabricated using the triangular lattice 2-D PLAs. The fabrication method and sensor structure was the same as that reported in our previous studies [17,18], except that formation of the surface pattern on the absorber was achieved using photolithography. Figure 3 shows the fabrication procedure used in this study. Figure 3a shows device fabrication on a 6-inch Si substrate using a standard complementary metal oxide semiconductor (CMOS) process. The thermocouples consist of a series of p- and n-type polycrystalline silicon semiconductors. The absorber area is formed with a 1.5 μm thick SiO_2_ layer. Figure 3b shows the periodic lattice structure, which is formed only on the SiO_2_ layer of the absorber area using reactive ion etching (RIE). Figure 3c shows that 50/250 nm thick Cr/Au layers are then sputtered on the SiO_2_ layer. The 250 nm thick Au layer is sufficiently thick to prevent IR light penetration, so that the influence of Cr and SiO_2_ beneath the Au layer is negligible. An Al layer is inserted at the backside of the absorber area to prevent backside absorption by SiO_2_. The Si substrate is anisotropically etched using tetramethylammonium hydroxide (TMAH) through the etching holes to form the cavity under the IR absorber area, as shown in Figure 3d. The TMAH used in this procedure is doped with Si so that the reflective backside Al layer is not etched. Finally, a thermally isolated floating structure is completed, on which the square/triangular lattice 2-D PLA is formed.

**Figure 4:**
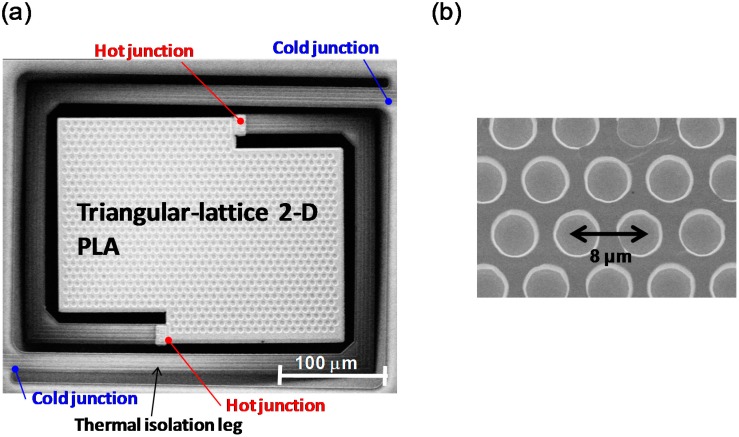
SEM image of (**a**) thermopile with triangular lattice 2-D PLA and (**b**) magnified image of the triangular lattice 2-D PLA.

Figure 4 shows scanning electron microscopy (SEM) images of the thermopile with the triangular lattice 2-D PLA. The magnified SEM image in Figure 4b shows a triangular lattice 2-D PLA with a period and diameter of 8.0 and 6.0 μm, respectively.

## 4. Measurements

**Figure 5:**
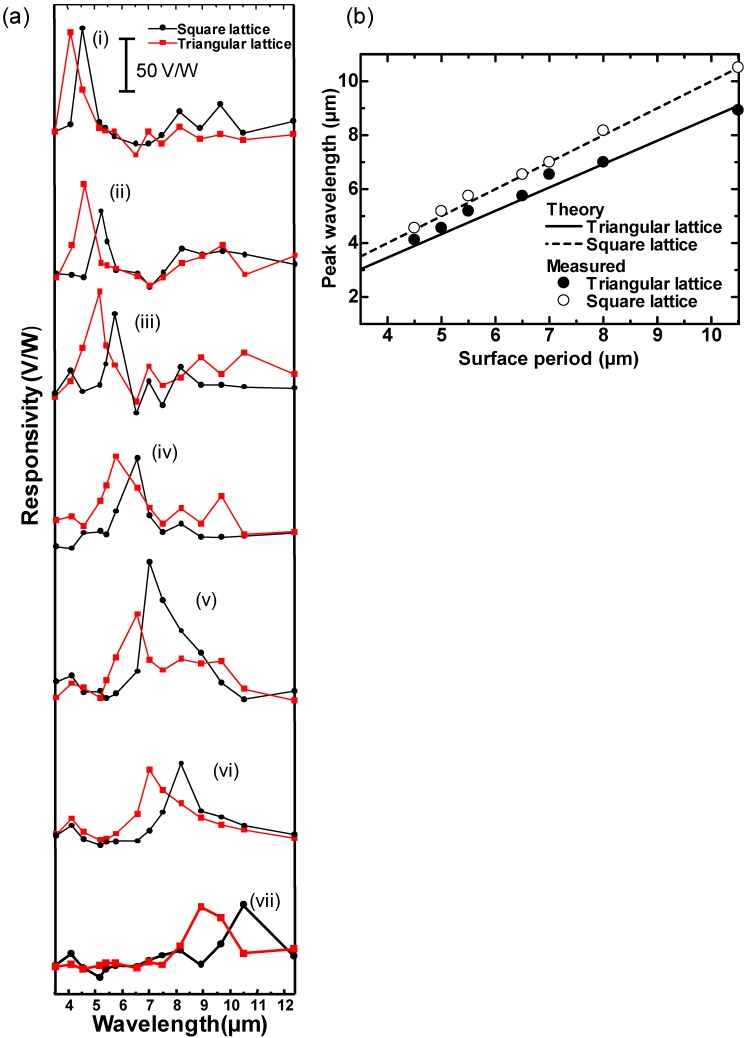
(**a**) Measured spectral responsivity, and (**b**) measured and calculated relation between the surface period and the detection wavelength for triangular and square lattice 2-D PLAs.

The spectral responsivity of the developed sensors was measured. The sensors were set in a vacuum chamber with a Ge window under a pressure of 1 Pa. The sensor was irradiated with IR radiation from a blackbody at a temperature of 1000 K and with an aperture diameter of 15.9 mm, which passed through narrow bandpass filters for selection of the evaluation wavelength. The distance between the sensors and the blackbody was 200 mm. The incidence angle was normal to the sensor and a pinhole was used to restrict the incident IR radiation to only the absorber area. The sensor output voltage was monitored by a computer. The responsivity was calculated as the ratio between the output voltage difference for the on and off states and the input power. The input power was calculated taking into account the measurement system parameters, absorber area, transmittance from the blackbody to the sensor through the atmosphere, narrow bandpass filters and the Ge window, and the spectral radiant emittance equation at the evaluated wavelength, as previously reported [16,17]. The measured diameters and periods of the surface structures were respectively: (i) 3.0 and 4.5 μm; (ii) 4.0 and 5.0 μm; (iii) 4.0 and 5.5 μm; (iv) 4.0 and 6.5 μm; (v) 6.0 and 7.0 μm; (vi) 6.0 and 8.0 μm; and (vii) 6.0 and 10.5 μm. The depth of the periodic lattice structures was fixed at 1.5 μm for all sensors. Figure 5a shows the measured spectral responsivity. Figure 5b shows the measured and theoretical dependence of the peak wavelength on the surface period. The spectral responsivity of the square lattice 2-D PLAs is also shown in Figure 5a,b for comparison. The measured results for the square lattice, except for structure (iii) and (v), were taken from our previous report [17]. 

Figure 5a,b show that wavelength-selective detection was also realized for the triangular lattice 2-D PLAs, and the absorption wavelengths for the triangular lattice 2-D PLAs were shorter than those for the square lattice 2-D PLAs. 

## 5. Discussion

Figure 5a,b demonstrate that the measured peak wavelengths correspond well with the theoretical investigations outlined in Section 2. We have previously demonstrated both numerically and experimentally [16,17] that the period is the main parameter that determines the absorption wavelength, whereas the depth and diameter have negligible impact. The ratio between the diameter and the period is most effective for obtaining sufficient absorption [26]. Therefore, these results demonstrate that the main detection wavelength can be controlled according to the primitive reciprocal lattice vector. Hence, the absorption of the 2-D PLA is attributed to the 2-D lattice surface plasmon resonance, which can be interpreted as Wood’s anomaly or Fano resonance [27]. The results obtained here will provide fundamental information on the design flexibility of wavelength-selective uncooled IR sensors using two types of 2-D lattice structures. The bandwidth is also important for practical applications and can be tuned using the ratio between the period and the diameter of the dimples [16].

## 6. Conclusions

Control of the detection wavelength was investigated for 2-D PLA lattice structures for wavelength-selective uncooled IR sensors. Thermopiles with triangular lattice 2-D PLAs were developed using standard CMOS-MEMS technology. Theoretical calculations and the measured spectral responsivity demonstrate that the detection wavelength for triangular lattice 2-D PLAs is shorter than that for square lattice 2-D PLAs, and the detection wavelength is determined by the primitive reciprocal lattice vector of the 2-D lattice structure. Precise control of the detection wavelength is required for practical applications and 2-D lattice control of the detection wavelength will facilitate expansion of the design flexibility. The results obtained here could be applied to other types of uncooled IR sensors such as bolometers or silicon-on-insulator diodes [28,29].
